# Single Nucleotide Polymorphisms in Vitamin D Receptor Gene Affect Birth Weight and the Risk of Preterm Birth: Results From the “Mamma & Bambino” Cohort and A Meta-Analysis

**DOI:** 10.3390/nu10091172

**Published:** 2018-08-27

**Authors:** Martina Barchitta, Andrea Maugeri, Maria Clara La Rosa, Roberta Magnano San Lio, Giuliana Favara, Marco Panella, Antonio Cianci, Antonella Agodi

**Affiliations:** 1 Department of Medical and Surgical Sciences and Advanced Technologies “GF Ingrassia”, University of Catania, via S. Sofia, 87, 95123 Catania, Italy; martina.barchitta@unict.it (M.B.); andreamaugeri88@gmail.com (A.M.); mariclalarosa@gmail.com (M.C.L.R.); robimagnano@gmail.com (R.M.S.L.); giuliana.favara@gmail.com (G.F.); 2 Department of General Surgery and Medical Surgical Specialties, University of Catania, Via S. Sofia, 78, 95123 Catania, Italy; mpanella@unict.it (M.P.); acianci@unict.it (A.C.)

**Keywords:** pregnancy, vitamin D, gestational duration, birth cohort

## Abstract

The effect of vitamin D receptor gene (VDR) polymorphisms on adverse pregnancy outcomes—including preterm birth (PTB), low birth weight and small for gestational age—is currently under debate. We investigated 187 mother-child pairs from the Italian “Mamma & Bambino” cohort to evaluate the association of maternal VDR polymorphisms—BsmI, ApaI, FokI and TaqI—with neonatal anthropometric measures and the risk of PTB. To corroborate our results, we conducted a meta-analysis of observational studies. For the FokI polymorphism, we showed that gestational duration and birth weight decreased with increasing number of A allele (*p* = 0.040 and *p* = 0.010, respectively). Compared to the GG and GA genotypes, mothers who carried the AA genotype exhibited higher PTB risk (OR = 12.049; 95% CI = 2.606–55.709; *p* = 0.001) after adjusting for covariates. The meta-analysis confirmed this association under the recessive model (OR = 3.67, 95%CI 1.18–11.43), and also pointed out the protective effect of BsmI polymorphism against the risk of PTB under the allelic (A vs. G: OR = 0.74; 95%CI 0.59–0.93) and recessive (AA vs. GG + AG: OR = 0.62; 95%CI 0.43–0.89) models. Our results suggest the association between some maternal VDR polymorphisms with neonatal anthropometric measures and the risk of PTB.

## 1. Introduction

Adverse pregnancy outcomes continue to be major Public Health problems in spite of improvements in health care [1,2,3]. Among these, preterm birth (PTB) represents the first cause of death among newborns and the second among children under five years [4]. Since the World Health Organization (WHO) estimated that 15 million of children prematurely born each year—it means more than one out of ten infants—novel strategies and guidelines should be designed and validated to help prevent PTB. The major risk factors of PTB are certainly maternal age, short inter-pregnancy interval, multiple gestation, drug abuse, smoking, vaginal dysbiosis and infections, low maternal pre-pregnancy weight or inadequate gestational weight gain [5,6,7,8]. However, the effect of genetic susceptibility has been also well recognized [9], with novel fetal and maternal genomic variants which in turn affect both intrauterine environment, pregnancy duration and fetal growth [10]. It has been estimated that approximately 32 million low birth weight (LBW) or small for gestational age (SGA) infants are born annually, with 96.5% of them in developing countries [11]. Approximately two-thirds of the risk of adverse pregnancy outcomes depend on maternal habits, with maternal nutrition playing a key role during the preconception and gestational periods [12]. However, uncovering the main genetic risk factors of adverse pregnancy outcomes remains one of the main challenges for Public Health, since they conferred about a third of this risk [13,14,15].

Overall, vitamin D is crucial for the maintenance of adult health [16] and its deficiency—especially during pregnancy—is associated with potential adverse outcomes for both mothers and children [17]. In humans, most of Vitamin D is provided by the endogenous cutaneous synthesis of pre-vitamin D3, which is derived from 7-dehydrocholesterol through the exposure to ultraviolet radiation [18]. The best sources of vitamin D are the flesh of fatty fish (i.e., salmon, tuna, and mackerel) and fish liver oils, while small amounts of vitamin D are found in beef liver, cheese, and egg yolks [19,20]. Vitamin D in these foods is primarily in the form of vitamin D3 [21]. In U.S.A. and Canada, fortified foods provide most of the vitamin D in the D3 form [19,22]. While Vitamin D3 represents almost 95% vitamin D serum levels [23], a less active form, known as Vitamin D2, is also provided by dietary sources and supplements [24]. Therefore, serum levels of 25-hydroxylated vitamin D2+D3 (25OHD) represent the vitamin D pool of the body. During pregnancy, the fetus is entirely dependent on maternal sources of vitamin D, which also regulates placental function [18]. Several observational studies showed that maternal vitamin D deficiency may influence mother and neonatal outcomes, including recurrent pregnancy losses, preeclampsia, gestational diabetes, PTB, LBW and SGA [25]. However, as concluded by recent systematic reviews and meta-analyses, it is currently not clear if adequate dietary intake and/or supplementation of vitamin D may reduce the risk of adverse pregnancy outcomes [25,26,27,28]. Inconclusive results could be partially explained by heterogeneity in study design, exposure variables, outcomes of interest, study setting and participants.

In this scenario, increasing interest concerns the effect of genetic variants affecting vitamin D metabolism and functions. Vitamin D activity is mediated by the vitamin D receptor (VDR), a nuclear receptor which acts as a high-affinity ligand-activated transcription factor [29]. VDR gene—located on the chromosome 12q12–14—is highly expressed in several human tissues including skin epithelium, osteoblasts and chondrocytes, muscles, cells from the immune system and placenta [30]. The ligand-bound VDR forms a heterodimer with nuclear retinoid X receptor (RXR) [31], which recognizes vitamin D response elements (VDRE) in the promoter regions of vitamin D target genes and recruits co-factors to modulate gene transcription [32]. Recently, some studies proposed the potential association between VDR polymorphisms and the risk of adverse pregnancy outcomes, such as PTB, LBW and SGA births [9,33,34,35,36,37,38,39,40,41,42]. In this area of research, BsmI (rs1544410), ApaI (rs7975232), FokI (rs2228570) and TaqI (rs731236) polymorphisms are the most commonly investigated: while TaqI and FokI consist of a single base change (A to G and G to A in exons 9 and 2, respectively), BsmI and ApaI are located in the last intron of the sequence and result from a single base change (G to A and A to C, respectively). However, evidence of an association between VDR polymorphisms and adverse pregnancy outcomes is currently weak and not convincing, with high heterogeneity across studies [43]. To explore the effect of preconception, perinatal and early life exposure on maternal and infant health—with particular focus on the interaction between epigenetic biomarkers of health and aging, diet and lifestyles [44,45,46,47,48]—we recently designed the prospective “Mamma & Bambino” study, which enrols mother-child pairs during pregnancy.

In the present study we used data and samples from this cohort to evaluate the association of maternal VDR polymorphisms (i.e., BsmI, ApaI, FokI and TaqI) with neonatal anthropometric measures and the risk of PTB, even considering dietary intake of vitamin D. Then, we carried out a systematic review evaluating the effect of VDR polymorphisms on PTB risk and on neonatal anthropometric measures. Finally, we corroborated our results by pooling them with those reported by previous studies through a meta-analysis.

## 2. Materials and Methods

### 2.1. Study Design

The “Mamma & Bambino” cohort is an ongoing Italian birth cohort designed to explore the effect of preconception, perinatal and early life exposure on maternal and infant health (further information can be found at http://www.birthcohorts.net). During the prenatal genetic counselling, at gestational week 4–20 (mean = 16 weeks), pregnant women referred to the Azienda Ospedaliera Universitaria “Policlinico-Vittorio Emanuele”, Catania (Italy) were invited to participate. This study was carried out in accordance with the Declaration of Helsinki and the protocol was approved by the ethics committee of the involved institution. All subjects were fully informed of the purpose and procedures and gave written informed consent. In this cohort, information on sociodemographic and lifestyle data are collected by trained epidemiologists using a structured questionnaire. Educational level is classified as low (primary school, i.e., ≤8 years of school) and high (high school education or greater, i.e., >8 years of school). Women are also classified as employed or unemployed (including students and housewives). Smoking status is classified as no smoking (including ex-smokers) and current smoking. Pre-pregnancy body mass index (BMI) is calculated as weight (kg) divided by height (m^2^), based on criteria from the WHO [49]. According to the Institute of Medicine (IOM) recommendations, we define adequate gestational weight gain (GWG) as follows: 12.5–18 kg (underweight), 11.5–16 kg (normal weight), 7–11.5 kg (overweight), and at least 5–9 kg (obese) [50]. Type of delivery, intrauterine foetal death, congenital malformations and plurality are also recorded at delivery. Biological samples are collected from both the mothers (peripheral blood) and the children (amniotic fluid and cord blood). Furthermore, a two-year follow-up is conducted to collect information on mother-child lifestyle and health status.

In the current analysis we included mother-child pairs with complete data on sociodemographic characteristics, lifestyle and vitamin D intake, pregnancy outcomes and maternal VDR genotype distributions. Primary outcomes were gestational duration and PTB, defined as spontaneous delivery before 37 weeks. Secondary outcomes were birth weight and length; birthweight was classified as LBW (birthweight < 2.5 kg) and macrosomia (birthweight ≥ 4.0 kg); birthweight for gestational age was defined as SGA, AGA or LGA according to sex-specific national reference charts [51]. Mother-child pairs with pre-existing medical conditions (i.e., autoimmune and/or chronic diseases), pregnancy complications (i.e., preeclampsia, hypertension and diabetes), pre-term induced delivery or caesarean section, intrauterine foetal death, plurality and congenital malformations were all excluded.

#### 2.1.1. VDR Genotyping

Maternal DNA was extracted from peripheral blood samples using QIAamp DNA Mini Kit according to the manufacturer protocol (Qiagen, Milan, Italy). VDR polymorphisms were genotyped using the following commercially available TaqMan SNP Genotyping Assays (Applied Biosystem, Foster City, CA, USA): ApaI rs7975232 (C_28977635_10), TaqI rs731236 (C_2404008_10), BsmI rs1544410 (C_8716062_10), FokI rs2228570 (C_12060045_20). All reactions were performed in triplicate on the QuantStudio™ 7 Flex Real-Time PCR System (Applied Biosystem, Foster City, CA, USA) deploying conditions set by the manufacturer. Allele determination was carried out using QuantStudio™ 7 Flex System Software (Applied Biosystem, Foster City, CA, USA).

#### 2.1.2. Assessment of Vitamin D Intake

Vitamin D intake was assessed by a validated 95-item semi-quantitative Food Frequency Questionnaire (FFQ) as described elsewhere [52]. In brief, women were asked to report the frequency of consumption (twelve categories from “almost never” to “two or more times a day”) and portion size (small, medium or large) of each food item, using and indicative photograph atlas. Food intakes were calculated by multiplying the frequency of consumption with the daily portion size of each food item. Vitamin D intake was calculated using the USDA Nutrient Database (http://ndb.nal.usda.gov/) adapted to the Italian food consumption. The use of multimineral/multivitamin supplements containing vitamin D was recorded, but the vitamin D intake was based only on food sources, as the FFQ was not designed to ascertain the quantification of vitamin D intake by supplementation.

#### 2.1.3. Statistical Analyses

Statistical analyses were conducted using SPSS software (IBM Corp. Released 2013. IBM SPSS Statistics for Windows, Version 22.0. Armonk, NY, USA). Descriptive statistics were used to characterize the study population, using frequency, mean and standard deviation (SD), or median and interquartile range (IQR). The Chi-square test was performed to determine if geno-type distributions in mothers with full-term delivery were deviated from the Hardy-Weinberg Equilibrium (HWE). Prior to analysis, the normal distribution of all variables was checked using the Kolmogorov-Smirnov test. Based on skewed distribution, comparisons of maternal and infant quantitative variables across VDR genotypes were analysed using the Mann-Whitney U test or the Kruskal-Wallis test. Categorical variables were compared using the Chi-squared test. Linear and binary regression models were applied to evaluate the associations of VDR polymorphisms with primary and secondary outcomes, using the non-mutated genotypes as reference. Regression models were adjusted for age, smoking, educational level, employment status, pre-gestational BMI, GWG, vitamin D intake, use of vitamin D supplements, type of delivery and parity. Post-hoc statistical power analysis was performed using Epi Info™ software (version 7; CDC, Atlanta, GA, USA). All statistical tests were two-sided, and *p* values < 0.05 were considered statistically significant.

### 2.2. Systematic Review and Meta-Analysis

#### 2.2.1. Search Strategy

Two of the Authors carried out a systematic literature search in the PubMed-Medline and Web of Science databases to identify relevant epidemiological studies, investigating the association of BsmI, ApaI, FokI and TaqI polymorphism with neonatal anthropometric measures and incidence of PTB, LBW and SGA births. The search strategy comprised the terms (Vitamin D receptor OR VDR) AND (variation OR polymorphism OR mutations OR SNP) AND (Preterm Birth OR birthweight OR birth weight OR birth length OR Low Birth Weight OR Intrauterine Growth Retardation OR Fetal Growth Retardation OR Small for Gestational Age). The databases were searched from inception to February 2018 without language restriction; abstracts and unpublished studies were not included. Moreover, the reference lists from selected articles, including relevant review papers, were searched to identify all appropriate studies. The preferred reporting items for systematic reviews and meta-analysis (PRISMA) guidelines were followed.

#### 2.2.2. Selection Criteria

Two of the Authors independently assessed the retrieved articles and any inconsistencies were resolved through discussion. Studies included were consistent with the following criteria: (i) observational studies or randomized control trials (RCTs) (ii) on pregnant women of any gestational age (iii) without pregnancy complications, (iv) focusing on the association of FokI, ApaI, TaqI and BsmI VDR polymorphisms with PTB, LBW, and SGA. Moreover, (v) studies were selected if they provide sufficient information on the numbers or genotype frequencies in cases and controls in order to estimate odds ratios (ORs) and 95% confidence intervals (95% Cis). By contrast, the exclusion criteria were as follow: (i) systematic reviews or meta-analyses; (ii) abstracts and unpublished studies; (iii) studies with insufficient or lack of data to estimate ORs and 95% CIs, after attempting to contact the corresponding authors via e-mail; (iv) investigating the association with other VDR polymorphisms (v) or with other adverse pregnancy outcomes; (vi) studies with no control group.

#### 2.2.3. Study Selection and Data Extraction

Two of the Authors independently extracted the following information: first Author’s last name, year of publication, country where the study was performed, ethnicity and number of participants, sample type, phenotype of the cases evaluated, genotyping method, genotype distributions in cases and controls, and *p*-values for HWE in controls. If additional data were needed, the Authors of retrieved articles were contacted. Primary outcome was PTB since lack of data and inconsistency of reported outcomes avoided the quantitative analysis for birthweight, birth length, LBW and SGA.

#### 2.2.4. Procedures of Meta-Analysis

Strength of association between VDR polymorphisms and PTB was estimated as ORs (95% CIs) under the allelic model (2 vs. 1), the dominant model (22 and 12 vs. 11) and the recessive model (22 vs. 11 and 12). The significance of pooled OR was determined by the Z test. Heterogeneity across studies was measured using the Q test, considering significant statistical heterogeneity as *p* < 0.1. As the Q test only indicates the presence of heterogeneity and not its magnitude, we also reported the *I*^2^ statistic, which estimates the percentage of outcome variability that can be attributed to heterogeneity across studies. An *I*^2^ value of 0% denotes no observed heterogeneity, whereas, 25% is “low”, 50% is “moderate” and 75% is “high” heterogeneity [53]. We also estimated the between-study variance using tau-squared (t) statistics [54]. According to heterogeneity across studies, we used the fixed-effects model (Mantel-Haenszel method) when heterogeneity was negligible or the random-effects models (DerSimonian-Laird method) when heterogeneity was significant. The presence of publication bias was investigated by Begg’s test and Egger’s regression asymmetry test [55,56]. Except for the Q test, *p* < 0.05 was considered statistically significant, and all tests were 2-sided. All statistical analyses were performed using the Review Manager software (Version 5.3. Copenhagen: The Nordic Cochrane Centre, the Cochrane Collaboration, 2014).

## 3. Results

### 3.1. “Mamma & Bambino” Cohort

From the “Mamma & Bambino” cohort, 187 women aged 15–50 years (median = 37 years) were enrolled at a median gestational age of 16 weeks (IQR = 4). According to pre-gestational BMI and GWG we identified 30.9% and 27.1% who exhibited reduced or excessive GWG, respectively. In general, gestational duration was 39 weeks (IQR = 2) and 55.1% of deliveries were natural. Median birth length and weight were 50.0 cm (IQR = 2) and 3.2 Kg (IQR = 0.6), respectively. Approximately 8% of new-borns were underweight while 7.5% was diagnosed with macrosomia. According to sex-specific national reference charts [51], most of new-borns were AGA (80.2%), while the proportion of SGA and LGA were 8% and 11.8%, respectively. Only 10.7% of women reported the use of multimineral/multivitamin supplements containing vitamin D. Notably, vitamin D intake was not associated with neonatal anthropometric measures nor with PTB risk (Table 1). Table 1 also shows the comparison between mothers with PTB (*n* = 17; 9%) and full-term delivery (*n* = 170, 91%). No differences were evident for maternal socio-demographic characteristics, pre-gestational BMI, GWG, use of vitamin D supplements and lifestyle. As expected, PTB new-borns exhibited lower birth length (47.5 cm vs. 50.0 cm; *p* < 0.001) and weight (2.44 kg vs. 3.2 kg; *p* < 0.001), with a higher proportion of underweight compared to full-term new-borns (58.8% vs. 2.9%; *p* < 0.001).

**Figure 1:**
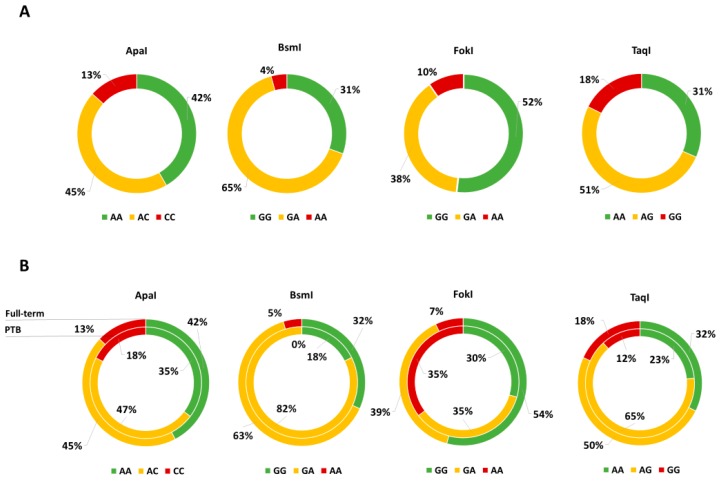
(**A**) Genotype Distribution of vitamin D receptor gene (VDR) Polymorphisms and (**B**) comparison between preterm births (PTB, inner ring) and full-term births (outer ring).

Figure 1A shows the distributions of VDR genotypes in the study population; notably, only FokI and TaqI VDR polymorphisms were in HWE. The comparison of neonatal anthropometric measures across TaqI genotypes showed that birth weight increased with increasing number of G allele (AA = 3.2 kg vs. AG = 3.2 kg vs. GG = 3.4; *p* = 0.020). However, we failed in confirming this difference after adjusting for covariates. The comparison of maternal and neonatal characteristics across FokI genotypes showed that gestational duration and birth weight decreased with increasing number of A allele (*p* = 0.040 and *p* = 0.010, respectively). Accordingly, the proportion of PTB increased with increasing number of A allele (GG = 5.2% vs. AG = 8.3% vs. AA = 33.3%; *p* = 0.001), whereas no statistically significant differences were reported for BsmI, ApaI and TaqI (Figure 1B). In line with this evidence, we demonstrated that the risk of PTB increased with the number of A allele both in the age-adjusted (OR = 3.010; 95% CI = 1.457–6.215; *p* = 003) and in the multivariable-adjusted models (OR = 4.015; 95% CI = 1.649–9.771; *p* = 0.002). Particularly, compared to the GG and GA genotypes, mothers who carried the AA genotype exhibited a higher PTB risk both in the age-adjusted (OR = 7.389; 95% CI = 2.308-23.660; *p* = 0.001) and in the multivariable-adjusted models (OR = 12.049; 95% CI = 2.606–55.709; *p* = 0.001).

### 3.2. Systematic Review

#### 3.2.1. Study Characteristics

**Figure 2:**
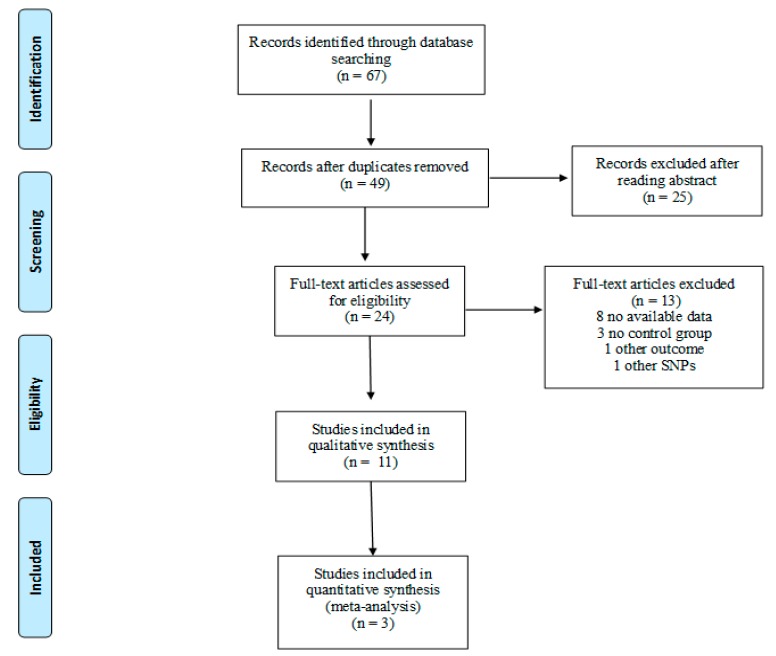
Flow diagram of study selection.

The detailed steps of study selection are given as a PRISMA flow diagram in Figure 2. A total of 67 articles were retrieved from the databases and 18 duplicates were excluded. After reading titles and/or abstracts, 25 articles were excluded while 24 underwent full-text screening. Based on selection criteria we excluded 13 studies, whereas 11 studies were included in the systematic review. No RCTs focusing on the association of VDR polymorphisms with PTB, LBW, and SGA were found and thus, only observational studies were included.

A total of 5 studies were from European countries [9,36,38,40,42], 4 from America [33,35,37,41], and 1 from Asia [34] and Australia [39], respectively. Overall, sample sizes ranged from 189 to 615 mothers and from 90 to 506 infants, respectively. The most reported outcome was PTB (*n* = 4) [9,33,34,36], while 3 studies investigated neonatal anthropometric measures using birth weight [37], LBW [35] or SGA [41] as primary outcome. Two studies collected both maternal and cord blood samples [34,36], while 5 and 3 studies genotyped VDR polymorphisms in cord blood [38,39,40,41,42] or maternal blood samples [9,33,37], respectively; only Workalemahu et al. collected placental samples [35]. Given that the majority analysed more than one polymorphism, FokI was analysed by 7 studies [33,34,35,36,37,39,41], BsmI by 8 studies [9,34,36,38,39,40,41,42], ApaI by 5 studies [9,34,36,37,39,40] and TaqI by 7 studies [9,34,36,37,38,39,40]. The most common genotyping method was restriction fragment length polymorphism analysis (RFLP) (*n* = 6) [34,36,37,38,39,40,42], followed by TaqMan SNP Genotyping Assays (*n* = 4) [9,33,37,41] and Sequenom MassARRAY (*n* = 1) [35].

#### 3.2.2. VDR Polymorphisms and Neonatal Anthropometric Measures

In 2011, Swamy and colleagues conducted a prospective study on 615 pregnant women, evaluating the effect of 38 VDR polymorphisms on several birth outcomes. In brief, they showed that 8 of 38 SNPs examined—including ApaI—were significantly associated with birth weight in black but not in white women [37]. In the same year, Silvano and colleagues studied 97 pre-pubertal singleton children from 0 to 12 years to assess clinical and biochemical phenotypes that better characterize SGA children who failed to achieve postnatal catch-up growth. At the baseline, they did not observe difference in BsmI and FokI genotype distributions across categories of birth weight for gestational age [41]. Consistently with our results, the study by Workalemahu et al.—investigating the effect of placental VDR polymorphisms on birth size in 506 mother-child pairs—demonstrated that birth weight decreased with increasing number of A allele of the FokI polymorphism [35].

#### 3.2.3. VDR Polymorphisms and PTB Risk

Manzon and colleagues genotyped VDR polymorphisms in both maternal and cord blood samples of 33 PTB and 98 full-term delivery [36]. In line with our results, they concluded that women who carried the A allele of FokI were at higher risk of PTB than those who carried the G allele. By contrast, women who carried the T allele of TaqI polymorphism exhibited a lower risk of PTB [36] compared to those with non-mutated allele [36]. However, using a logistic regression model they observed that only maternal FokI variant was associated with the risk of PTB. More recently, the study by Javorsky and colleagues—comparing104 women with PTB to 85 with full-term delivery—confirmed that maternal FokI polymorphism was associated with a higher risk of PTB [33]. Next, Rosenfeld and colleagues added to this knowledge, demonstrating that the proportion of women with PTB decreased with increasing number of A allele of maternal BsmI polymorphism, after adjusting for some confounders [34]. Interestingly, among women with previous history of spontaneous miscarriage, the risk of PTB was higher if their newborns carried the non-mutated allele of BsmI or the mutated allele of ApaI [34].

#### 3.2.4. Meta-Analyses of the Association between VDR Polymorphisms and PTB Risk

**Figure 3:**
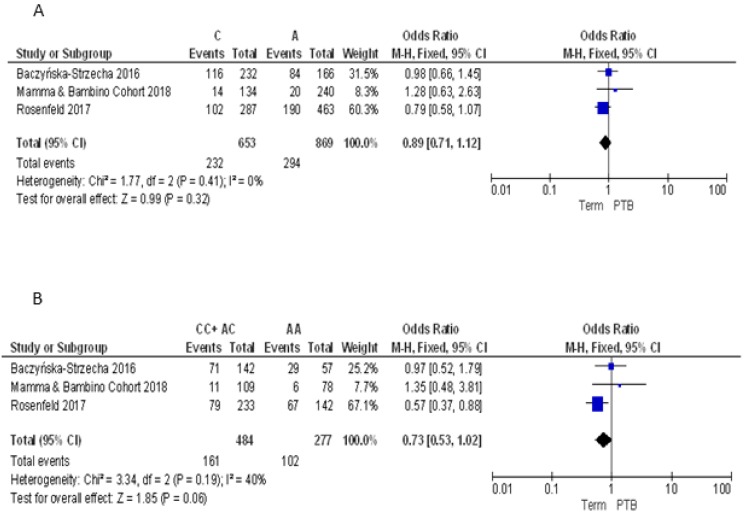

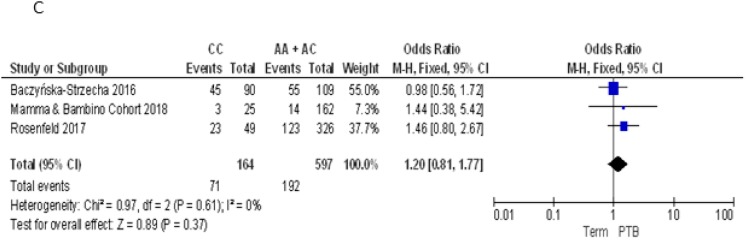
Forest plots of the association between ApaI polymorphism and preterm birth under the (**A**) allelic, (**B**) dominant and (**C**) recessive models.

As demonstrated by post-hoc statistical power analysis, only the study of the association between FokI polymorphism and PTB reached a statistical power of at least 80%, with a significance level of 0.05. Thus, to summarize evidence about the association between maternal VDR polymorphisms and PTB, we pooled our results with those reported by three previously published articles [9,33,34] (Table 2). For the ApaI polymorphism, we pooled our results with those reported by Baczyńska-Strzecha et al. and Rosenfeld et al. [9,34]. The Q-test and *I*^2^ statistics showed no significant heterogeneity across studies under the allelic (*p* = 0.41; *I*^2^ = 0%), dominant (*p* = 0.19; *I*^2^ = 40%) and recessive (*p* = 0.61; *I*^2^ = 0%) models. Using the fixed effect model, the meta-analysis showed no association of ApaI with PTB under the allelic model (C vs. A: OR = 0.89, 95%CI 0.71–1.12), dominant (CC + AC vs. AA: OR = 0.73, 95%CI 0.53–1.2) and recessive model (CC vs. AA + AC: OR = 1.20, 95%CI 0.81–1.77) (Figure 3).

**Figure 4:**
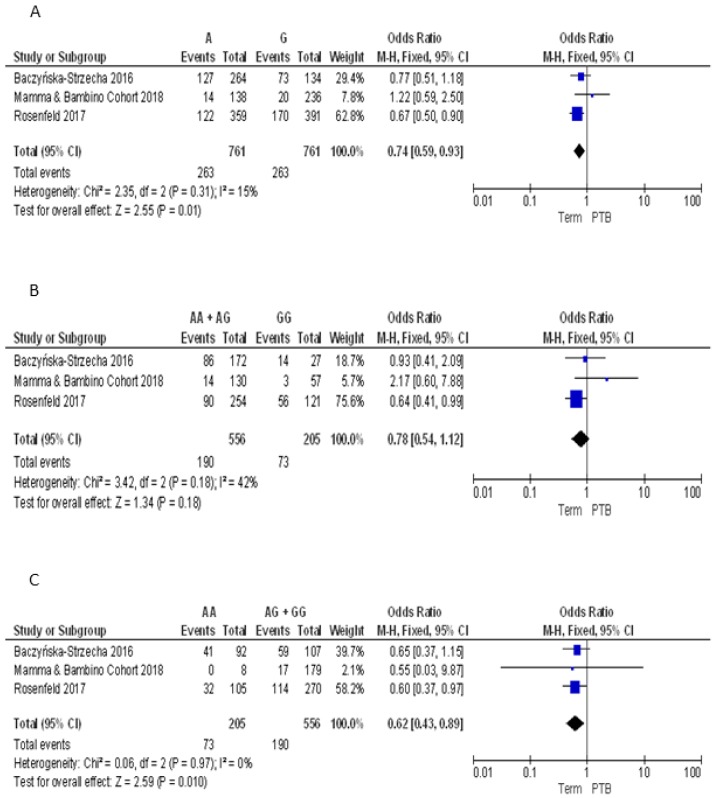
Forest plots of the association between BsmI polymorphism and preterm birth under the (**A**) allelic, (**B**) dominant and (**C**) recessive models.

For the BsmI polymorphism, we pooled our results with those reported by Baczyńska-Strzecha et al. and Rosenfeld et al. [9,34]. The Q-test and *I*^2^ statistics showed no significant heterogeneity across studies under the allelic (*p* = 0.31; *I*^2^ = 15%), dominant (*p* = 0.18; *I*^2^ = 42%) and recessive (*p* = 0.97; *I*^2^ = 0%) models. Using the fixed effect model, the meta-analysis showed a significant negative association with PTB under the allelic (A vs. G: OR = 0.74, 95%CI 0.59–0.93) and recessive (AA vs. GG + AG: OR = 0.62, 95%CI 0.43–0.89) models. By contrast, no statistically significant association was evident under the dominant model (AA + AG vs. GG: OR = 0.78, 95%CI 0.54–1.12) (Figure 4).

**Figure 5:**
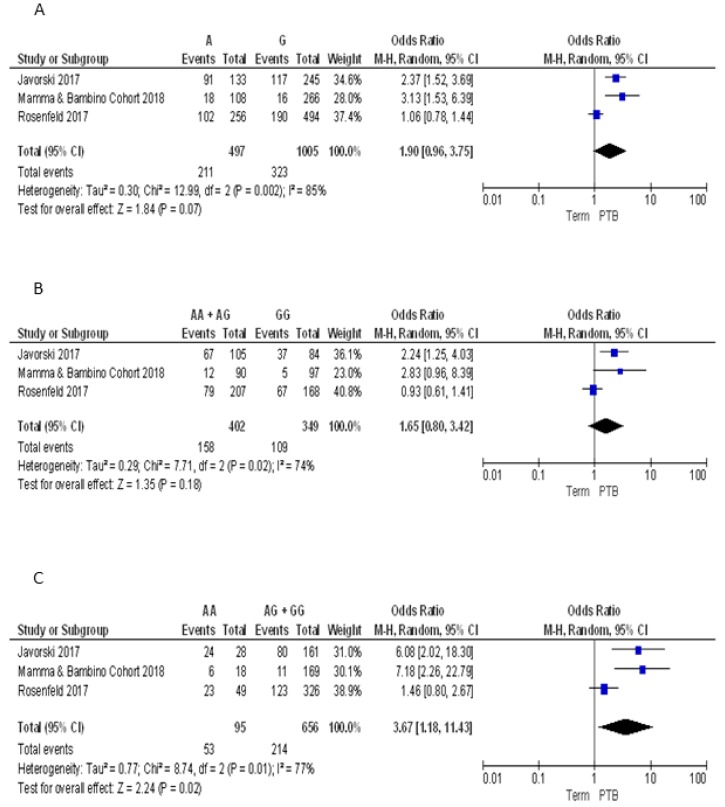
Forest plots of the association between FokI polymorphism and preterm birth under the (**A**) allelic, (**B**) dominant and (**C**) recessive models.

For the FokI polymorphism we pooled our results with those reported by Javorski et al. and Rosenfeld et al. [33,34]. The Q-test and *I*^2^ statistics showed significant heterogeneity across studies under the allelic (*p* = 0.002; *I*^2^ = 85%), dominant (*p* = 0.02; *I*^2^ = 74%) and recessive models (*p* = 0.001; *I*^2^ = 77%). Using the random effect model, the meta-analysis showed a significant association with PTB under the recessive model (AA vs. GG + AG: OR = 3.67, 95%CI 1.18–11.43). By contrast, pooled ORs under the allelic (A vs. G: OR = 1.90, 95%CI 0.96–3.75) and dominant (AA + AG vs. GG: OR = 1.65, 95%CI 0.80–3.42) models were not statistically significant (Figure 5).

**Figure 6:**
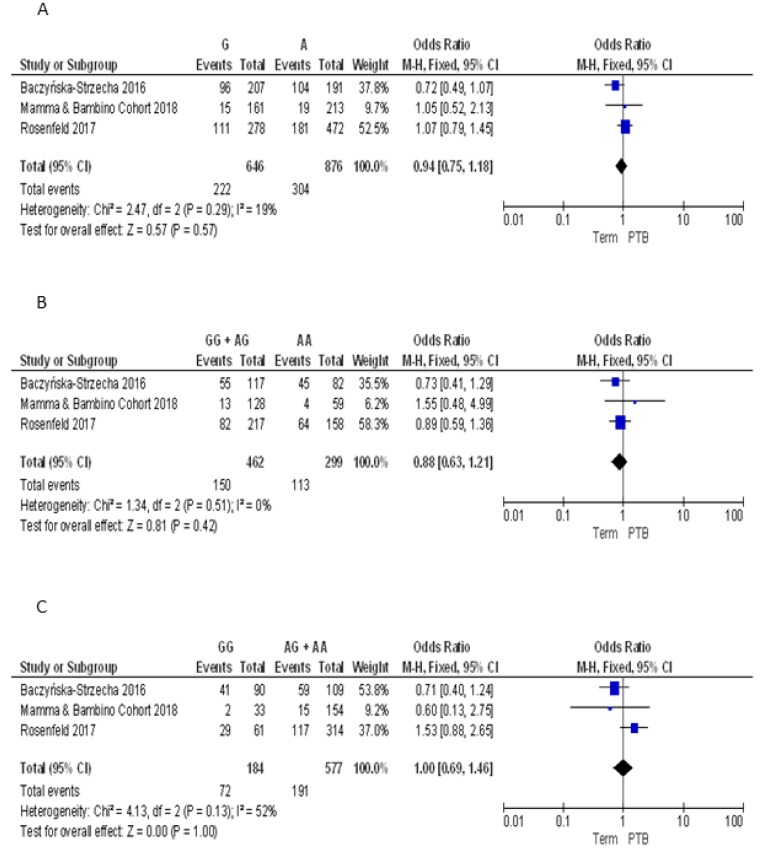
Forest plots of the association between TaqI polymorphism and preterm birth under the (**A**) allelic, (**B**) dominant and (**C**) recessive models.

For the TaqI polymorphism we pooled our results with those reported by Baczyńska-Strzecha et al. and Rosenfeld et al. [9,34]. The Q-test and *I*^2^ statistics showed no significant heterogeneity across studies under the allelic (*p* = 0.29; *I*^2^ = 19%), dominant (*p* = 0.51; *I*^2^ = 0%) and recessive (*p* = 0.13; *I*^2^ = 55%) models. Using the fixed effect model, the meta-analysis showed no significant association with PTB under the allelic (G vs. A: OR = 0.94, 95%CI 0.75–1.18), dominant (GG + AG vs. AA: OR = 0.88, 95%CI 0.63–1.21), and recessive (GG vs. AA + AG: OR = 1.00, 95%CI 0.69–1.46) models (Figure 6). Overall, we found no evidence of publication bias for meta-analyses of ApaI (Begg’s *p* = 0.74; Egger’s *p* = 0.89), BsmI (Begg’s *p* = 0.70; Egger’s *p* = 0.19), FokI (Begg’s *p* = 0.87; Egger’s *p* = 0.81), and TaqI (Begg’s *p* = 0.42; Egger’s *p* = 0.24).

## 4. Discussion

In the present study, we used data from the “Mamma & Bambino” cohort to evaluate the effect of VDR polymorphisms on neonatal anthropometric measures and on the risk of PTB. Interestingly, we observed that maternal FokI polymorphism affected both gestational duration and birth weight, which decreased with increased number of the mutated allele (A). This is consistent with Workalemahu and colleagues that demonstrated, for the first time, that birth weight decreased with increasing number of A allele [35]. In line with reduced gestational duration, we also demonstrated that the risk of PTB increased with increasing number of A allele both in the age-adjusted and in the multivariable-adjusted models. Notably, compared to mothers with GG or GA genotypes, mothers who carried the AA genotype exhibited a 12-fold increased risk of PTB, independent of socio-demographic characteristics (age, educational level, employment status), lifestyle (smoking, pre-gestational BMI, GWG), vitamin D intake, use of vitamin D supplements, type of delivery and parity. A similar risk was also observed by Manzon et al. [36] and Javorsky et al. [33], while Rosenfeld and colleagues failed in demonstrating this association. When we pooled our results with those reported by Javorsky et al. and Rosenfeld et al. [33,34], we demonstrated a significant positive association between FokI polymorphism and PTB under the recessive model. By contrast, Rosenfeld and colleagues [34] also demonstrated that the proportion of women with PTB decreased with increasing number of mutated allele (A) of maternal BsmI polymorphism. Although we failed in demonstrating this association, the meta-analysis confirmed the protective effect of BsmI against PTB under the allelic and recessive models. Results about the effect of TaqI polymorphism on PTB risk are currently inconclusive: while Manzon and colleagues proposed that the mutated allele conferred a lower PTB risk [36], we did not confirm this association by pooling our results with those reported by Baczyńska-Strzecha et al. and Rosenfeld et al. [9,34]. However, since we observed that birth weight increased with increasing number of mutated alleles, it cannot be completely excluded the protective effect of TaqI polymorphism on foetal growth and development. A prospective study on 615 pregnant women, conducted by Swamy and colleagues, evaluated the effect of 38 VDR polymorphisms on several birth outcomes. Since 8 of 38 SNPs examined—including ApaI—significantly affected birth weight in black but not in white women, the Authors concluded that strength of association may depend on ethnicity, proposing a partial explanation for the observed racial disparity in several pregnancy outcomes [37]. However, non-significant results in white women may be the result of the substantially smaller sample for compared to the black group. Nevertheless, comparison between studies should be interpreted with caution due to the high heterogeneity across studies in terms of design, sample size and type, ethnicity, geographical diversity, sun exposure, maternal habits and outcomes of interest, that may account for discrepancies in the obtained results [9,33,34].

The mechanistic link between VDR expression and foetal outcome is still unclear. Calcitriol is the major active ligand of VDR. The ligand-bound VDR forms a heterodimer with nuclear retinoid X receptor (RXR) [31], which recognizes vitamin D response elements (VDRE) in the promoter regions of vitamin D target genes and recruits co-factors to modulate gene transcription [32]. However, vitamin D can also exert rapid non-genomic effects, probably via VDR located within the plasma membrane [57]. This rapid pathway works via specific enzymes, such as protein kinase C and mitogen-activated protein kinase [57], which regulate cell proliferation and differentiation, invasive processes and apoptosis. Although several studies suggest that vitamin D system—including VDR, its ligands and the metabolizing enzymes—plays a key role in innate immunity and implantation [58,59,60,61,62], the functional effects of VDR and its allelic variants in pregnancy are not yet clarified. The main weakness of our study was the relatively small sample size and the statistical power—with a low number of PTB infants—that raises the need of future analyses on the ongoing “Mamma & Bambino” cohort to confirm the observed associations. Although we acknowledge that further stratification by ethnicity should be provided—the “Mamma & Bambino” cohort consisted of Caucasian women—we established the effect of VDR polymorphisms on PTB risk in the context of previous studies investigating this association in medium-large populations that included different racial and ethnic groups. However, due to inconsistency of reported outcomes, we cannot perform a meta-analysis of the association between VDR polymorphisms and neonatal anthropometric measures. Indeed, only three studies investigated the effect on neonatal anthropometric measures using birth weight [37], LBW [35] or SGA [41] as primary outcome. Furthermore, the collection of additional data regarding other anthropometric measures should be included in future studies. Another weakness was that we did not analysed VDR polymorphisms in infant samples, not being able to assess the contribution of foetal VDR gene on adverse pregnancy outcomes. In fact, as reported by Rosenfeld et al., the risk of PTB could be higher in new-borns carrying the non-mutated BsmI or the mutated ApaI allele [34]. Moreover, the influence of unmeasured variables, such as sun exposure, maternal habits and serum vitamin D levels cannot be completely excluded. Since the FFQ did not quantify vitamin D from nutritional supplements and fortified foods, we did not have a precise measure of vitamin D intake status. However, we reported that the use of vitamin D supplements containing is not common among women from the “Mamma % Bambino” cohort and no association with PTB risk was evident. Furthermore, the measurement of 25OHD would be useful to evaluate whether there was a relationship between serum levels and dietary intake, including vitamin D supplements. In addition, it should be recognized that, in the present study, other variables such as poverty status, mid-pregnancy immune and growth-related factors, recently associated with PTB prediction in women with and without preeclampsia [63], have not been included. Notably, our study was conducted in a cohort of the women in Sicily, southern Italy and a recent systematic review reports that despite high levels of sunshine, maternal hypovitaminosis D during pregnancy is prevalent in sunny Mediterranean region where optimal vitamin D levels are expected [64]. Reasons for this phenomenon may rely on different factors that can determine vitamin D deficiency.

Finally, in future studies the effects of other single nucleotide polymorphisms in genes encoding for key components of the vitamin D metabolism pathway—such as genes involved in cholesterol synthesis (DHCR7), hydroxylation (CYP2R1, CYP24A1), and vitamin D transport (GC) that influence vitamin D status [64,65] should be addressed. We acknowledge that unmeasured variables in the current analysis limit our ability to draw conclusions on the association of maternal VDR polymorphisms with neonatal anthropometric measures and the risk of PTB in our cohort. Despite such limitations, the strictly selection criteria ruled out potential confounders such as pre-existing medical conditions, pregnancy complications, pre-term induced delivery or caesarean section, intrauterine foetal death, plurality and congenital malformations. Moreover, extensive data collection enabled to adjust our results for socio-demographic factors, lifestyle, pre-gestational BMI and GWG, and vitamin D dietary intake. Additional researches, including observational prospective studies and clinical trials, are recommended to establish the role of vitamin D and related factors in pregnancy, and to develop and validate effective preventive strategies against adverse outcomes.

## 5. Conclusions

In conclusion, we provide novel evidence and a meta-analysis about the effect of VDR polymorphisms on birth weight and gestational duration, identifying a group of women at risk of PTB.

## Figures and Tables

**Table 1:** Population characteristics and comparison between preterm and full-term births.

Characteristics	Study Population (*n* = 187)	PTB (*n* = 17)	Full-Term (*n* = 170)	*p*-Value
**Age, years ^a^**	37.0 (4)	37.0 (5)	38.0 (4)	0.648
**Gestational age at enrolment, weeks**	16.0 (4)	16.0 (4)	16.0 (4)	0.691
**Educational level (% low-medium)**	13.9%	5.9%	14.7%	0.316
**Employment status (% employed)**	55.6%	47.1%	56.5%	0.456
**Smoking (% current smokers)**	18.3%	5.8%	19.5%	0.165
**Pre-gestational nutritional status**				
Underweight	8.1%	11.8%	7.7%	0.522
Normal weight	65.6%	58.8%	66.3%	
Overweight	17.2%	11.8%	17.8%	
Obese	9.1%	17.6%	8.3%	
**GWG, kg ^a^**	12.0 (7)	11.0 (10)	12.0 (6.9)	0.630
**GWG classification**				
Reduced	30.9%	35.3%	30.5%	0.903
Adequate	42%	41.2%	42.1%	
Excessive	27.1%	23.5%	27.4%	
**Vitamin D intake, µg/day ^a^**	3.7 (3.5)	3.7 (4.3)	3.1 (3.6)	0.808
**Vitamin D supplements (% users)**	10.7%	5.9%	11.2%	0.501
**Gestational duration, weeks**	39.0 (2)	35.5 (2)	39.0 (2)	<0.001
**Sex (% male)**	50.3%	47.1%	50.6%	0.781
**Birth weight, kg ^a^**	3.2 (0.6)	2.44 0.5)	3.2 (0.6)	<0.001
**Birth length, cm ^a^**	50.0 (2)	47.5 (4)	50.0 (2)	<0.001
**Type of delivery**				
Natural	55.1%	47.1%	55.9%	0.486
Caesarean section	44.9%	52.9%	44.1%	
**Underweight (%)**	8%	58.8%	2.9%	<0.001
**Macrosomia (%)**	7.5%	0%	8.2%	0.219
**Weight for gestational age**				
SGA	8%	5.9%	8.2%	0.283
AGA	80.2%	70.6%	81.2%	
LGA	11.8%	23.5%	10.6%	

^a^ Data reported as median (IQR). Abbreviations: IQR, interquartile range; PTB, preterm birth; GWG, gestational weight gain; SGA, small for gestational age; AGA, adequate for gestational age; LGA, large for gestational age.

**Table 2:** Characteristics of studies included in the meta-analysis.

Authors	Country	Study Design	Ethnicity	Sample Size	Sample	SNPs	Genotyping Method
**Baczyńska-Strzecha et al., 2016** [9]	Poland	Case-control	Caucasian	199	Maternal blood	ApaI BsmI TaqI	TaqMan Assay
**Javorski et al., 2018** [33]	Brazil	Case-control	Mixed	189	Maternal blood	FokI	TaqMan Assay
**Rosenfeld et al., 2017** [34]	Israel	Case-control	Mixed	375	Maternal and fetal blood	ApaI BsmI FokI TaqI	RFLP
**Mamma & Bambino Cohort, 2018**	Italy	Prospective cohort	Caucasian	187	Maternal blood	ApaI BsmI FokI TaqI	TaqMan Assay

Abbreviations: SNP, single nucleotide polymorphism.

## References

[1] Chiavaroli V., Castorani V., Guidone P., Derraik J.G., Liberati M., Chiarelli F., Mohn A. (2016). Incidence of infants born small- and large-for-gestational-age in an italian cohort over a 20-year period and associated risk factors. Ital. J. Pediatr..

[2] Lawn J.E., Blencowe H., Pattinson R., Cousens S., Kumar R., Ibiebele I., Gardosi J., Day L.T., Stanton C. (2011). Stillbirths: Where? When? Why? How to make the data count?. Lancet.

[3] Goldenberg R.L., Culhane J.F., Iams J.D., Romero R. (2008). Epidemiology and causes of preterm birth. Lancet.

[4] Howson C.P., Kinney M.V., McDougall L., Lawn J.E. (2013). Born too soon: Preterm birth matters. Reprod. Health.

[5] Vogel J.P., Chawanpaiboon S., Moller A.B., Watananirun K., Bonet M., Lumbiganon P. (2018). The global epidemiology of preterm birth. Best Pract. Res. Clin. Obstet. Gynaecol..

[6] Vitale S.G., Marilli I., Rapisarda A.M., Rossetti D., Belluomo G., Iapichino V., Stancanelli F., Cianci A. (2014). Cellular and biochemical mechanisms, risk factors and management of preterm birth: State of the art. Minerva Ginecol..

[7] Giunta G., Giuffrida L., Mangano K., Fagone P., Cianci A. (2012). Influence of lactoferrin in preventing preterm delivery: A pilot study. Mol. Med. Rep..

[8] Pino A., Giunta G., Randazzo C.L., Caruso S., Caggia C., Cianci A. (2017). Bacterial biota of women with bacterial vaginosis treated with lactoferrin: An open prospective randomized trial. Microb. Ecol. Health Dis..

[9] Baczyńska-Strzecha M., Kalinka J. (2016). Influence of apa1 (rs7975232), taq1 (rs731236) and bsm1 (rs154410) polymorphisms of vitamin d receptor on preterm birth risk in the polish population. Ginekol. Pol..

[10] York T.P., Eaves L.J., Neale M.C., Strauss J.F. (2014). The contribution of genetic and environmental factors to the duration of pregnancy. Am. J. Obstet. Gynecol..

[11] Black R.E. (2015). Global prevalence of small for gestational age births. Nestle Nutr. Inst. Workshop Ser..

[12] Chavarro J.E., Rich-Edwards J.W., Rosner B.A., Willett W.C. (2006). Iron intake and risk of ovulatory infertility. Obstet. Gynecol..

[13] Lieberman E., Gremy I., Lang J.M., Cohen A.P. (1994). Low birthweight at term and the timing of fetal exposure to maternal smoking. Am. J. Public Health.

[14] Pietrantoni M., Knuppel R.A. (1991). Alcohol use in pregnancy. Clin. Perinatol..

[15] Fowles E.R. (2004). Prenatal nutrition and birth outcomes. J. Obstet. Gynecol. Neonatal. Nurs..

[16] Ji J.L., Muyayalo K.P., Zhang Y.H., Hu X.H., Liao A.H. (2017). Immunological function of vitamin D during human pregnancy. Am. J. Reprod. Immunol..

[17] Kiely M., Hemmingway A., O’Callaghan K.M. (2017). Vitamin D in pregnancy: Current perspectives and future directions. Ther. Adv. Musculoskelet. Dis..

[18] Holick M.F. (2011). Vitamin D: A D-lightful solution for health. J. Investig. Med..

[19] Institute of Medicine (2011). Dietary Reference Intakes for Calcium and Vitamin D.

[20] U.S. Department of Agriculture, Agricultural Research Service (ARS) USDA National Nutrient Database for Standard Reference. https://www.ars.usda.gov/northeast-area/beltsville-md-bhnrc/beltsville-human-nutrition-research-center/nutrient-data-laboratory/docs/usda-national-nutrient-database-for-standard-reference/.

[21] Ovesen L., Brot C., Jakobsen J. (2003). Food contents and biological activity of 25-hydroxyvitamin D: A vitamin D metabolite to be reckoned with?. Ann. Nutr. Metab..

[22] Calvo M.S., Whiting S.J., Barton C.N. (2004). Vitamin D fortification in the United States and Canada: Current status and data needs. Am. J. Clin. Nutr..

[23] Holick M.F. (2003). Vitamin D: A millenium perspective. J. Cell. Biochem..

[24] Norman A.W. (2012). The history of the discovery of vitamin D and its daughter steroid hormone. Ann. Nutr. Metab..

[25] Harvey N.C., Holroyd C., Ntani G., Javaid K., Cooper P., Moon R., Cole Z., Tinati T., Godfrey K., Dennison E. (2014). Vitamin D supplementation in pregnancy: A systematic review. Health Technol. Assess..

[26] De-Regil L.M., Palacios C., Lombardo L.K., Peña-Rosas J.P. (2016). Vitamin D supplementation for women during pregnancy. Sao Paulo Med. J..

[27] Pérez-López F.R., Pasupuleti V., Mezones-Holguin E., Benites-Zapata V.A., Thota P., Deshpande A., Hernandez A.V. (2015). Effect of vitamin D supplementation during pregnancy on maternal and neonatal outcomes: A systematic review and meta-analysis of randomized controlled trials. Fertil. Steril..

[28] Thorne-Lyman A., Fawzi W.W. (2012). Vitamin D during pregnancy and maternal, neonatal and infant health outcomes: A systematic review and meta-analysis. Paediatr. Perinat. Epidemiol..

[29] Strugnell S.A., Deluca H.F. (1997). The vitamin d receptor-structure and transcriptional activation. Proc. Soc. Exp. Biol. Med..

[30] Wang Y., Zhu J., DeLuca H.F. (2012). Where is the vitamin D receptor?. Arch. Biochem. Biophys..

[31] Freedman L.P., Arce V., Perez Fernandez R. (1994). DNA sequences that act as high affinity targets for the vitamin D3 receptor in the absence of the retinoid x receptor. Mol. Endocrinol..

[32] Karras S.N., Wagner C.L., Castracane V.D. (2018). Understanding vitamin d metabolism in pregnancy: From physiology to pathophysiology and clinical outcomes. Metabolism.

[33] Javorski N., Lima C.A.D., Silva L.V.C., Crovella S., de Azêvedo Silva J. (2018). Vitamin D receptor (VDR) polymorphisms are associated to spontaneous preterm birth and maternal aspects. Gene.

[34] Rosenfeld T., Salem H., Altarescu G., Grisaru-Granovsky S., Tevet A., Birk R. (2017). Maternal-fetal vitamin D receptor polymorphisms significantly associated with preterm birth. Arch. Gynecol. Obstet..

[35] Workalemahu T., Badon S.E., Dishi-Galitzky M., Qiu C., Williams M.A., Sorensen T., Enquobahrie D.A. (2017). Placental genetic variations in vitamin D metabolism and birthweight. Placenta.

[36] Manzon L., Altarescu G., Tevet A., Schimmel M.S., Elstein D., Samueloff A., Grisaru-Granovsky S. (2014). Vitamin D receptor polymorphism foki is associated with spontaneous idiopathic preterm birth in an israeli population. Eur. J. Obstet. Gynecol. Reprod. Biol..

[37] Swamy G.K., Garrett M.E., Miranda M.L., Ashley-Koch A.E. (2011). Maternal vitamin D receptor genetic variation contributes to infant birthweight among black mothers. Am. J. Med. Genet. A.

[38] Lorentzon M., Lorentzon R., Nordström P. (2000). Vitamin D receptor gene polymorphism is associated with birth height, growth to adolescence, and adult stature in healthy Caucasian men: A cross-sectional and longitudinal study. J. Clin. Endocrinol. Metab..

[39] Tao C., Yu T., Garnett S., Briody J., Knight J., Woodhead H., Cowell C.T. (1998). Vitamin D receptor alleles predict growth and bone density in girls. Arch. Dis. Child..

[40] Suarez F., Zeghoud F., Rossignol C., Walrant O., Garabédian M. (1997). Association between vitamin d receptor gene polymorphism and sex-dependent growth during the first two years of life. J. Clin. Endocrinol. Metab..

[41] Silvano L., Miras M., Pérez A., Picotto G., Díaz de Barboza G., Muñoz L., Martin S., Sobrero G., Armelini P., Mericq V. (2011). Comparative analysis of clinical, biochemical and genetic aspects associated with bone mineral density in small for gestational age children. J. Pediatr. Endocrinol. Metab..

[42] Suarez F., Rossignol C., Garabédian M. (1998). Interactive effect of estradiol and vitamin D receptor gene polymorphisms as a possible determinant of growth in male and female infants. J. Clin. Endocrinol. Metab..

[43] Knabl J., Vattai A., Ye Y., Jueckstock J., Hutter S., Kainer F., Mahner S., Jeschke U. (2017). Role of placental VDR expression and function in common late pregnancy disorders. Int. J. Mol. Sci..

[44] Agodi A., Barchitta M., Quattrocchi A., Maugeri A., Vinciguerra M. (2015). Dapk1 promoter methylation and cervical cancer risk: A systematic review and a meta-analysis. PLoS ONE.

[45] Agodi A., Barchitta M., Quattrocchi A., Maugeri A., Canto C., Marchese A.E., Vinciguerra M. (2015). Low fruit consumption and folate deficiency are associated with line-1 hypomethylation in women of a cancer-free population. Genes Nutr..

[46] Barchitta M., Quattrocchi A., Maugeri A., Vinciguerra M., Agodi A. (2014). Line-1 hypomethylation in blood and tissue samples as an epigenetic marker for cancer risk: A systematic review and meta-analysis. PLoS ONE.

[47] Barchitta M., Quattrocchi A., Maugeri A., Canto C., La Rosa N., Cantarella M.A., Spampinato G., Scalisi A., Agodi A. (2017). Line-1 hypermethylation in white blood cell dna is associated with high-grade cervical intraepithelial neoplasia. BMC Cancer.

[48] Barchitta M., Maugeri A., Quattrocchi A., Agrifoglio O., Agodi A. (2017). The role of mirnas as biomarkers for pregnancy outcomes: A comprehensive review. Int. J. Genomics.

[49] (1995). Physical status: The use and interpretation of anthropometry: Report of a WHO expert committee. World Health Organ. Tech. Rep. Ser..

[50] Moore Simas T.A., Waring M.E., Sullivan G.M., Liao X., Rosal M.C., Hardy J.R., Berry R.E. (2013). Institute of medicine 2009 gestational weight gain guideline knowledge: Survey of obstetrics/gynecology and family medicine residents of the united states. Birth.

[51] Bertino E., Spada E., Occhi L., Coscia A., Giuliani F., Gagliardi L., Gilli G., Bona G., Fabris C., De Curtis M. (2010). Neonatal anthropometric charts: The Italian neonatal study compared with other European studies. J. Pediatr. Gastroenterol. Nutr..

[52] Agodi A., Barchitta M., Valenti G., Marzagalli R., Frontini V., Marchese A.E. (2011). Increase in the prevalence of the mthfr 677 tt polymorphism in women born since 1959: Potential implications for folate requirements. Eur. J. Clin. Nutr..

[53] Higgins J.P., Thompson S.G. (2002). Quantifying heterogeneity in a meta-analysis. Stat. Med..

[54] Higgins J.P., Green S. (2008). Cochrane Handbook for Systematic Reviews of Interventions. https://handbook-5-1.cochrane.org/.

[55] Begg C.B., Mazumdar M. (1994). Operating characteristics of a rank correlation test for publication bias. Biometrics.

[56] Egger M., Davey Smith G., Schneider M., Minder C. (1997). Bias in meta-analysis detected by a simple, graphical test. BMJ.

[57] Shin J.S., Choi M.Y., Longtine M.S., Nelson D.M. (2010). Vitamin D effects on pregnancy and the placenta. Placenta.

[58] Evans K.N., Bulmer J.N., Kilby M.D., Hewison M. (2004). Vitamin D and placental-decidual function. J. Soc. Gynecol. Investig..

[59] Stephanou A., Ross R., Handwerger S. (1994). Regulation of human placental lactogen expression by 1,25-dihydroxyvitamin d3. Endocrinology.

[60] Avila E., Díaz L., Barrera D., Halhali A., Méndez I., González L., Zuegel U., Steinmeyer A., Larrea F. (2007). Regulation of vitamin D hydroxylases gene expression by 1,25-dihydroxyvitamin d3 and cyclic amp in cultured human syncytiotrophoblasts. J. Steroid. Biochem. Mol. Biol..

[61] Evans K.N., Nguyen L., Chan J., Innes B.A., Bulmer J.N., Kilby M.D., Hewison M. (2006). Effects of 25-hydroxyvitamin d3 and 1,25-dihydroxyvitamin d3 on cytokine production by human decidual cells. Biol. Reprod..

[62] Murthi P., Yong H.E., Ngyuen T.P., Ellery S., Singh H., Rahman R., Dickinson H., Walker D.W., Davies-Tuck M., Wallace E.M. (2016). Role of the placental vitamin d receptor in modulating feto-placental growth in fetal growth restriction and preeclampsia-affected pregnancies. Front. Physiol..

[63] Jelliffe-Pawlowski L.L., Rand L., Bedell B., Baer R.J., Oltman S.P., Norton M.E., Shaw G.M., Stevenson D.K., Murray J.C., Ryckman K.K. (2018). Correction: Prediction of preterm birth with and without preeclampsia using mid-pregnancy immune and growth-related molecular factors and maternal characteristics. J. Perinatol..

[64] Karras S., Paschou S.A., Kandaraki E., Anagnostis P., Annweiler C., Tarlatzis B.C., Hollis B.W., Grant W.B., Goulis D.G. (2016). Hypovitaminosis D in pregnancy in the Mediterranean region: A systematic review. Eur. J. Clin. Nutr..

[65] Wang T.J., Zhang F., Richards J.B., Kestenbaum B., van Meurs J.B., Berry D., Kiel D.P., Streeten E.A., Ohlsson C., Koller D.L. (2010). Common genetic determinants of vitamin d insufficiency: A genome-wide association study. Lancet.

